# “Coming Home Is the Hardest Part”: An Interpretative Phenomenological Analysis of Sense Making in Military Postdeployment Reintegration

**DOI:** 10.1002/jcop.23178

**Published:** 2025-01-13

**Authors:** Tiffani N. Luethke, Herb L. Thompson, Gwendolynn Folk

**Affiliations:** ^1^ Department of Communication University of Nebraska at Kearney Kearney Nebraska USA; ^2^ School of Communication University of Nebraska at Omaha Omaha Nebraska USA

**Keywords:** deployment, interpretative phenomenological analysis (IPA), military service members and veterans (MSMVs), reintegration, trauma

## Abstract

The purpose of the present interpretative phenomenological analysis (IPA) study was to understand how military service members and veterans (MSMVs) make sense of their reintegration experiences following deployment. IPA provides the ability to gain a deeper understanding of a shared experience, or phenomenon, such as reintegration following deployment. Data collection involved semi‐structured interviews via Zoom. Participants included seven men and three women who met eligibility criteria. The present study provides important insights into how MSMVs make sense of their reintegration experiences following deployment through exploration of transitional challenges, transitional support aspects, and growth through deployment experiences, as well as the urgent need for comprehensive community‐based, growth‐focused initiatives to support their reintegration following deployment.

The most recent report from the US Department of Veterans Affairs (2022) states that 6146 veteran suicide deaths occurred in 2020 and, overall, suicide ranked as the 13th leading cause of death among veterans. In 2019, the rate of suicide deaths among veterans in the first 12 months of separation from service was 47.8 per 100,000 which represents the highest rate in 9 years (US Department of Veterans Affairs 2022). Also of note is the clear existence of increased suicidal ideation among service members. Research and reporting conducted by the Iraq and Afghanistan Veterans Affairs (2010) reported that, “In 2021, it was found that 9 [sic] percent of respondents had considered suicide before joining the military whereas 44% had considered suicide after joining the military” (Statista Search Department 2022). These alarming statistics illustrate the importance of research relating the experiences of military members as they transition from active deployment. The literature indicates the merit in this area however opportunities remain for those seeking to explore the sense these individuals make of their uniquely demanding experiences as military service members and veterans.

Current research suggests that communication challenges, role conflicts, and emotional turbulence often characterize the experiences of postdeployment military members as they reintegrate into the domestic and relational structures back *home* (Knobloch, Basinger, and Theiss 2018; Orazem et al. 2017; Parker et al. 2019). Moreover, the literature provides compelling documentation of these challenges (e.g., Chernichky‐Karcher and Wilson 2017; Gettings et al. 2019; Knobloch et al. 2013; Theiss & Knobloch 2013). However, few studies have investigated how the sensemaking among military members may assist in this complicated process of returning home. The challenges of returning active‐duty military service members has garnered considerable attention (Gettings et al. 2019; Orazem et al. 2017; Rossetto 2015; Sayer et al. 2011a). In this literature review, we seek to orient the reader to topics relevant to the central question of this study: How do military service members and veterans (MSMVs) make sense of the transition home following deployment?

## 1 Reintegration

Scholarly interest in MSMV reintegration has shown notable growth recently, with particular focus on Operation Enduring Freedom (OEF) and Operation Iraqi Freedom (OIF). This research is a response to the needs presented by veterans, active‐duty service members, and their families which require mental health, behavioral health services, and social services. Initiatives like the Wounded Warrior Project illustrate the beginning of efforts to provide systemic and adequate support for those with behavioral health needs during the reintegration process. An example of such work is a qualitative interpretive meta‐synthesis conducted by Tarbet, Moore and Alanazi (2021), which provided in‐depth analysis into eight studies that highlighted nine themes of reintegration, including fear of death, feelings of alienation, loss of collective, loss of control, loss of identity, loss of purpose, reliance on authority, resentment towards civilians, self‐isolation, and sense of duty. Insights from studies like this one contribute to our knowledge of reintegration issues to date.

Military reintegration has been defined as, “the postdeployment [sic] achievement of satisfactory levels of functioning at home, at work, in relationships, and in the community” (Sayer et al. 2011b, p. 662). The deployment itself introduces many emotional and relational challenges for military service members and their loved ones (Rossetto 2015) and the challenges presented in a return home have been found to be similarly difficult to navigate (Orazem et al. 2017). Theiss and Knobloch (2013) emphasize this importance when concluding, “Thus, understanding how military personnel engage in relational communication during reintegration can be important for helping military couples negotiate the transition effectively” (p. 1110). While the military does provide some resources to assist this process of reintegration (Gettings et al. 2019), it is still unclear how MSMVs make sense of this process.

The literature highlights a number of specific challenges related to the reintegration process for MSMV's. These range from posttraumatic stress (Gibbons et al. 2012), depression and relational distress (Knobloch et al. 2013), emotional disconnection (Theiss & Knobloch 2013), parenting stress (Chernichky‐Karcher and Wilson 2017), and community disconnection (Sayer et al. 2011a). These challenges can also manifest in risky behaviors such as substance abuse, emotional volatility, and relational neglect (Gettings et al. 2019). One concerning aspect of readjustment for service members is a common reluctance to request help due to concerns about the perception of other military members (Hoge et al. 2004; Katz et al. 2010). This “requesting help” stigma was particularly high among those most in need of mental health services (Hoge et al. 2004). Rossetto (2015) confirms the importance of reintegration‐coping resources to increase the success of re‐entry for MSMV's and their loved ones.

Relationships constitute an important component of the reintegration process. Knobloch, Basinger and Theiss (2018) offer some helpful insight into the challenges and changes that relationships, specifically romantic ones, undergo during reintegration. Postdeployment introduces challenges in relational *transitions* which are explained as those experiences when partner relationships are interrupted resulting in the co‐creation of new norms to relate and connect with one another. These transition periods have been identified as potential triggers for re‐evaluation in relational interdependence because of the impact it has on the certainty or uncertainty experienced by MSMVs and significant others during reintegration. Knobloch, Basinger and Theiss (2018) explain that these *transitions*, “by their very nature, spark questions about the status of the relationship and compel partners to restructure interdependence” (Knobloch, Basinger, and Theiss 2018, p. 54). Romantic partners and loved ones must abruptly navigate these new norms of relational interdependence while concurrently dealing with the unique stressors that accompany military deployment (Orazem et al. 2017). This adjustment often creates strain for the individual reintegrating as well as their significant others (Knobloch et al. 2013; Sayer et al. 2011a). Taken together, the task of relational adjustment during transition demonstrates a complex challenge for those involved (Chernichky‐Karcher and Wilson 2017; Katz et al. 2010).

## 2 Posttraumatic Growth (PTG)

PTG is a well‐documented phenomena among MSMVs (see for e.g., Mark et al. 2018). Specific findings related to PTG and the military include meaning‐making (Parrish‐Sprowl et al. 2014), posttraumatic stress (Tsai et al. 2015), psychosocial factors (Tsai and Pietrzak 2017), and satisfaction with life (Morgan et al. 2017), among many others. While experiences of PTG are established among MSMVs, specific findings related to cultural perspective taking, or the development of intercultural competency have not been widely explored in previous research. Conceptual work by Buechner (2020) proposed that the development of skills including “the ability to view situations from the perspective of others, or acquiring a less culturally‐dependent worldview, or intercultural competency” (p. 11) may assist with the transition from military to civilian life at the end of service through developing cultural agility. Oliva (2022) suggested that having a global mindset may assist veterans in transitioning to the civilian workforce. Yet to date, these skills have not been well‐documented among MSMVs following deployment experiences. In the present study, the specific themes of *Appreciation and Respect for Cultural Difference* and *Adapting to Cultural Difference* are unique and contribute new insights to the body literature.

## 3 Communication Strategies for Military Personnel

The field of communication provides a number of conceptual frameworks to better understand the experiences of MSMVs as they reintegrate. As was asserted earlier, service members face numerous unique challenges related to reintegration, in particular in their close relationships. Miscommunication during deployment and reintegration has been linked to increased frustrations and relational network breakdown (Hinojosa, Hinojosa, and Högnäs 2012). The communication patterns of returning military members have been examined in various relational dynamics from romantic partners (Knobloch, Basinger, and Theiss 2018), to adolescent children (Chernichky‐Karcher and Wilson 2017), and general family members (Gettings et al. 2019) during reintegration. Frequently, quality communication between veterans and their children was also found to lower spousal stress responses and increase deep engagement with children (Houston et al. 2013).

In examining the interactions of children and their military parents, Chernichky‐Karcher and Wilson (2017) identified patterns of communication that may facilitate or frustrate perceived family understanding for parents and their children during reintegration. Among other findings, they discovered that conformity‐driven communication patterns result in greater adolescent difficulties than do conversation‐focused communication patterns employed by postdeployment parents (Chernichky‐Karcher and Wilson 2017).

These findings, along with the other literature reviewed in this section, confirm the value of exploration into communication and relational dynamics in support networks for MSMVs as they reintegrate. While previous research provides an orientation to the relevance of communication patterns in the support of military personnel following deployment, there is opportunity for exploration into the sense‐making of MSMVs during the relational transitions experienced as part of reintegration (Boros and Erolin 2021; McCormack and Ell 2017). The purpose of the present study was to understand how MSMVs make sense of their reintegration experiences following deployment, with a particular focus on the challenges they faced, aspects that were helpful, and their own experiences of growth.

## 4 Methodology

We used a qualitative approach, involving interpretative phenomenological analysis (IPA) to better understand how MSMVs make sense of their reintegration experiences following deployment. IPA was selected as an appropriate method for this study given its ability to offer a “flexible and versatile approach to understanding people's experiences” (Tuffour 2017, p. 5). IPA is a methodological stream within the qualitative tradition of phenomenology that examines the rich descriptions of participants regarding the relationships, emotions, and sensemaking connected to their experiences of a stated phenomenon (Smith and Nizza 2022; Smith et al. 2009). This method is matched for this present study because of its focus on understanding, “people's lived experience and how they make sense of it in the context of their personal and social worlds” (Smith and Nizza 2022, p. 3). This research aims to understand how MSMVs make sense of their reintegration context and process as expressed through their communicative and relational experiences. IPA was utilized by the research team to organize the research questions, analysis, and description of results. In summary, “IPA is a qualitative research approach committed to the examination of how people make sense of their major life experiences” (Smith, Flowers, and Larkin 2022, p. 1). What makes IPA unique is the concentration it applies to the sensemaking of participants. We selected IPA because it positions this study as a supplement to the existing literature on this topic by providing an analysis of meaning‐making (Smith et al. 2009). We obtained approval for this study from the University of Nebraska at Kearney's Institutional Review Board, and we provided informed consent to all participants. To protect participants' identities, we utilized pseudonyms for names of people included in this study.

## 5 Participants

It is recommended that IPA studies include a sample ranging from 3 to 10 participants given its aim to “find a reasonably homogeneous sample, so that, within the sample, we can examine convergence and divergence in some detail” (Smith et al. 2009, p. 3). Participants included seven men and three women who met eligibility criteria (i.e., 19 years of age or older; military service member or veteran, have been deployed on active duty, and consented to participate). This group included veterans and military service members returning from a variety of deployment experiences, including wars and combats as well as noncombats (e.g., located in the US), short tours, and expeditionary units (which typically included several locations). The length of single deployments also varied from approximately 1 month to more than 2 years. However, 8 of 10 participants reported at least one deployment lasting 6 months or longer. We acknowledge that the wide range of deployment experiences (i.e., combat vs. noncombat zones, length of time, location of deployment) may be a limitation of this study. For instance, participants experiencing deployment in active combat zones will face many additional challenges (e.g., trauma, lack of sleep, night terrors, posttraumatic stress, and more) versus those in noncombat zones. In the present study, the focus on reintegration following a term of deployment offers insights about challenges, support aspects, and growth related to reintegration following a term of active duty service, typically far from home (*N* = 9) and away from one's family members (*N* = 9).

Participants ranged in age from 26 to 79 (*M* = 41.40; SD = 16.11). At the time of this study, all participants reported that they were employed or retired, and the majority of participants (90%) reported a household income of $50,000 or greater. Furthermore, 50% of the participants were enrolled as students (full or part time). Half of our participants (50%) were married or living with a significant other, 40% were single and never married, and 10% were separated or divorced. Of note, all female participants were single and never married, which may be a limitation of this study. Nearly all of our participants (90%) reported that they had completed some college, with 30% reporting that they had completed a graduate degree. Participants self‐selected their race and ethnicity with 90% identifying as White and 10% identifying as Hispanic/Latino. The number and length of deployments varied by participant. Military affiliation(s) and individual ranks are reported in Table 1, along with locations and dates of deployments. Locations of deployment included a wide range of regions and countries.

**Table 1:** Participant demographics, military affiliation, rank, and deployment details.

Name	Gender	Current marital status	Age	Branch of military affiliation(s)	Current military rank	Dates and locations of deployments**
Aaron	Male	Married or living with partner	52	Air Force	E‐7/MSgt (Ret)	12/90 ‐ 05/91 Saudi Arabia 01/97 ‐ 04/97 Turkey 02/98 ‐ 05/98 Turkey 02/99 ‐ 05/99 Italy 01/05 ‐ 05/05 Japan
Daniel	Male	Separated or divorced	26	The Army National Guard	E‐4/CPL	03/17‐ 03/18 Kuwait and Syria
David	Male	Married or living with partner	54	The Army National Guard	E‐7/SFC (Ret)	01/03 ‐ 10/04 United States
Jake	Male	Married or living with partner	34	Marine Corps	E‐7/GySgt	11/11 ‐ 05/12 Afghanistan 05/13 ‐ 11/13 Japan & Australia 11/17 ‐ 05/18 Japan
Jessica	Female	Single, never married	37	The Army	O‐4/MAJ	09/06 ‐ 11/07 Iraq 04/09 ‐ 06/10 Afghanistan
Joan	Female	Single, never married	79	Air Force	O‐5/Lt Col (Ret)	10/67 ‐ 10/68 Vietnam 08/70 ‐ 08/71 Canada 07/81 ‐ 07/84 Germany
Matthew	Male	Married or living with partner	35	Marine Corps	E‐8/MSgt	09/04 ‐ 04/05 Iraq 11/06 ‐ 11/06 Japan, South Korea, Thailand, Philippines, Cambodia, and Australia 06/13 ‐ 02/14 Portugal, Spain, Greece, Libya, Croatia, Jordan, Djibouti, Yemen, Oman, and Bahrain
Megan	Female	Single, never married	31	Navy, Reserves	E‐6/PO1	06/14 ‐ 12/14 Naval Ship 03/18 ‐ 12/18 United States 05/20 ‐ 05/21 Naval Ship
Spencer	Male	Married or living with partner	38	Marine Corps, National Guard	E‐4/*	08/04 ‐ 03/05 Iraq 02/06 ‐ 10/06 Iraq
Thomas	Male	Single, never married	28	The Army	N/A*	2013 Afghanistan 2015 Korea

*Note:* Military rank designations of E denote Enlisted, NCO denote Noncommissioned Officer, W denote Warrant Officer, and O denote Commissioned Officer. For a full list and explanation of military ranks, please visit: https://www.va.gov/vetsinworkplace/docs/em_rank.html.

* Participant did not disclose this information.

** Category presented as self‐reported by participants.

It is worth noting that all participants, with the exception of David, served at least one period of active‐duty deployment overseas. David's experience also differed in that he was able to live with his family during deployment due to the proximity of his deployment location to his home. However, David's accounts of reintegration paralleled other participants in many ways, including experiences of relational turbulence which resulted in a divorce (though he was remarried at the time of this study).

### 5.1 Procedures

Participants were recruited via social media, word of mouth, and through snowball sampling. Data collection involved up to two interviews with each participant via Zoom. The first author acted as the primary investigator and conducted all interviews, including the Intercultural Development Inventory debriefing and coaching sessions. We used a semi‐structured interview protocol in alignment with the recommendations from Smith et al. (2022) for conducting IPA research to help solicit rich and in‐depth responses about the participants' experiences. The present research occurred during the COVID‐19 pandemic which restricted our ability to meet and interact with participants in face‐to‐face settings. Therefore, all participants were interviewed using Zoom. An unanticipated benefit of this approach appeared to be an increased level of comfort for participants because they were able to share their stories from the convenience of their own space.

All three authors contributed to the data analysis process, using an IPA approach, which was facilitated by MAXQDA analysis software. Specifically, we used an iterative process to analyze the interview transcripts and reflective prompts. Our analysis involved line‐by‐line coding of our data to identify experiential statements, which later became personal experiential themes (PETs [Smith et al. 2022]). In essence, experiential statements relate directly to the participants' sensemaking of their reintegration experiences (Smith et al. 2022). We met on five separate occasions to discuss experiential statements and find agreement regarding the final PETs. We determined that data saturation occurred when our study could be replicated (O'Reilly and Parker 2012; Walker 2012), additional interviews would not be likely to reveal new experiential statements, and further coding of our data was not feasible (Fusch and Ness 2015; Guest, Bunce, and Johnson 2006).

### 5.2 Validation Strategies

Our validation efforts included peer review, member checking, collaboration, triangulation, and rich thick description. According to Creswell and Poth (2018) member checking ensures that interpreted findings accurately represent participant perspectives. In the present study, member checking occurred by inviting feedback from participants about the study's findings. Additionally, a peer reviewer, with expertize in the area of veterans and mental health, provided yet another layer of validation by examining the research process, methods, data interpretation, and findings (Creswell and Poth 2018). Collaboration as a form of validation (Creswell and Miller 2000) occurred by having multiple investigators involved throughout the research process. The comparison of reflective prompts and participant interviews (Creswell and Poth 2018; Deegan 2007) along with multiple participant perspectives (Guest, 2014) provided triangulation. Finally, we achieved rich thick description by providing detailed accounts of our participants' experiences (Creswell and Miller 2000; Denzin 1989).

## 6 Findings

### 6.1 Contextual Information

Our participants were diverse in terms of their age, length of time in the military, specific roles and responsibilities, branch of military and level of distinction as well as the number of times deployed and location of deployments. Despite these differences, we found a few points of commonality within our participants' backgrounds. For instance, all participants discussed an initial enthusiasm for joining the military. Many expressed a sense of, as Jessica said, “doing something worthwhile.” While some participants joined the military because of a perceived sense of patriotism (e.g., being motivated by the events of 9/11), others joined as a means to pay for college, and still others joined from a sense of generational obligation or pride. It is worth noting that anticipation of deployment was affected by whether participants joined the military pre‐ versus post‐9/11. Specifically, our more mature participants (i.e., having joined pre‐9/11) typically had not considered the possibility of deployment until it occurred. In contrast, those who joined post 9/11, acknowledged the possibility of deployment upon joining.

Most participants acknowledged the possibility of deployment when they initially joined the military while a small number did not really consider the possibility of deployment until it actually occurred. Other common threads resulting from deployment included the sense of real mortality (i.e., life and death scenarios), working tiresome shifts (often 12–16 h per day) with little sleep and limited time off. Many participants discussed experiences of sleep deprivation related to drills or real danger (such as mortars impacting near or even inside the walls of their military base) and the difficulty of living under constant threat resulting in mental fatigue along with many other symptoms. Despite these challenges, most participants related that the hardest part of deployment occurred when reintegrating into postdeployment life.

### 6.2 Personal Experiential Themes

In all, our analysis revealed three main categories of PETs framing our understanding of how these participants made sense of their reintegration experiences, including (1) transitional challenges, (2) transitional support aspects, and (3) growth through deployment. Within each category we identified clusters of experiential statements that led to the development of PETs. In the present study, we discuss those PETs most relevant when seeking to understand how participants made sense of their reintegration experiences. A full list of the PETs and example experiential statements are provided in Table 2.

**Table 2:** Personal experiential themes and experiential statement examples.

	Personal experiential theme	Experiential statement examples
Transitional challenges	“Coming home is the hardest part”	We spent months and months training me to go back to our job but spent so little time [to] reintegrate back home. (Daniel)
	Relationship turbulence	So just living back with your family can be a challenge if you don't do it slowly, because as people, they've learned to survive without you. (Matthew)
	“It does affect you”: Impacts on mental health	I have trouble sleeping…But I just you can't just go out and just turn it off…I can't shut it off. (Daniel)
	Unhealthy coping	Doing a lot of bad things, really…I went to alcohol…not necessarily a good thing to do…and I'm not going to be the first vet to tell you that a lot of us have gone in that direction. (Aaron)
Transitional support aspects	Communication	Identify any issues and problems firsthand to talk about them before they boil over to cause bigger problems. (Jake)
	Having a shared experience with other vets	The one thing that helped was…talking to other vets that had been there…because we spoke all the same language (Aaron)
	Establishing a support network	Having a support network even during a deployment is important. (Jessica)
	Creating stability and routine	It's like you get right back into your routine…gym, eat, sleep, hang out with the family, whatever that routine looks like inside the household, that is, you got to. (Matthew)
	Asking for help	Don't be afraid to ask for help…and take anything that's offered for help. Take it into consideration. (Daniel)
	Productive coping	You're either going through it and you take it. And you're gonna get through it, and you'll eventually help someone else out with it. (Thomas)
Growth through deployment experiences	New perspective on and for life	So I have a you know, a respect for life. I don't know if I thought about it before ever going on a deployment. (Matthew)
	Empathy	I can understand. It makes me a little more empathetic [to] others and what they're going through (Thomas)
	Appreciation and respect for cultural difference	Instead of seeing them as an enemy, see him as, we can still see him as an enemy. But they're no different than you and me. They got a family. (Daniel)
	Adapting to cultural difference	Just go somewhere and figure out how to be part of their culture, it's really not too hard…You adjust to that culture, at least somewhat…That's it for any culture, just be respectful (Megan)
	Resilience and adaptation	I think I became a lot more resilient out there, a lot tougher. I don't let stuff bother me near as much as I used to. (Megan)
	Leadership	I think I've just grown overall as a professional but especially as a leader and how to lead people and handle subordinates. (Megan)

### 6.3 Transitional Challenges

As the review of literature demonstrated, challenges related to reintegration following a term of deployment are well‐documented. Therefore, it is not surprising that this investigation of MSMV's sensemaking related strongly with the themes of, (1) “Coming home is the hardest part,” (2) Relationship turbulence, (3) “It does affect you”: Impacts on mental health, and (4) Unhealthy coping.

#### 6.3.1 “Coming Home Is the Hardest Part”

Nearly all participants discussed the difficulty of fully transitioning after their deployment experiences. Descriptions like being stuck in “war mode” and not being able to “shut it off” help explain such experiences. Daniel shared, “The hard part has just started. It's coming home, reintegrating, and learning to survive in your home.” He went on to explain how he tried to return to normalcy following deployment, “But you're just you're on edge,” adding, “We spent months and months training me to go back to our job, but spent so little time [to] reintegrate back home.” Many participants openly discussed the struggles of coming home, including having nightmares and experiencing extreme sensitivity to fireworks, sirens, and large crowds, thoughts of suicide, feelings of anger or callousness, reliance on alcohol, self‐isolation from family and friends, and feeling a sense of loss and guilt. Spencer shared his own experience of losing a fellow soldier and the subsequent guilt, “But having somebody that I fought with at my side–that I've kind of lost that. And it's made a very large impact. And my son is actually named after one of my good friends that got killed. I figured that was the least I can do. I have a lot of survivor's remorse.”

Participants also shared other costs related to deployment, such as the loss of a marriage or the loss of one's health. Nearly all participants discussed the strain of deployment and of returning after deployment on intimate relationships. Some participants experienced a divorce firsthand, which contributed to the difficulty of transitioning after deployment. David shared, “I got divorced and all the kids were living with their mom. I was able to see them, but that was…a dysfunctional type thing in a way where, you know, [I] don't have a family, where I have them, but you know, to go see the kids, I got to go pick them up and take them to wherever…”

#### 6.3.2 “It Does Affect You”: Impacts on Mental Health

Additionally, participants discussed how they made sense of having limited access to the resources required. They also disclosed experiences of being misdiagnosed or undiagnosed, which often resulted in a lack of proper treatment. Aaron shared his experience, “That first time, you know, there was no help, OK? I know I suffered from PTSD, but I got evaluated and declined.” As part of the larger mental health discussion, many participants noted that awareness of and access to mental health resources is better today than it was in the past. Yet, greater suicide prevention efforts must be prioritized, as Aaron also shared, “You've heard the story of…a veteran commits suicide because of the transitioning. We need to communicate that, we need to evaluate that. They need to evaluate maybe even a little bit different on posttraumatic stress syndrome and all that stuff. Because I'll tell you. For that transitioning period coming back, it's not easy and never is easy, even especially coming back from a combat zone is never, never, never easy.” Additionally, several participants discussed being disbanded from their units following their return from deployment, which often required relocation and separation from individuals with whom they had established trusting relationships. Some participants also ended their military service and transitioned into civilian positions following deployment. In both cases, participants shared how these experiences contributed to feelings of disconnection and isolation, and, in some cases, made it difficult to locate and use resources to help support their transition.

Like Aaron, a majority of participants discussed struggling with mental health in some way after returning from deployment. Another barrier to accessing mental healthcare services was the notion that asking for help demonstrated weakness. Daniel shared, “Suddenly, so many people don't reach out for help because a lot of times we think reaching for help is weakness. But in reality, it actually makes us stronger, actually asking for the help.” In addition to the transitional challenges that participants shared as part of their sense‐making process, they also discussed aspects which helped facilitate their reintegration process.

### 6.4 Transitional Support Aspects

Helpful aspects that assisted participants in making sense of their reintegration experiences focused on common components of connection to and communication with others. Participants cited examples, such as taking time to transition, learning to take things in stride and being slow to anger, establishing a routine or sense of control, focusing on personal goals or purpose–especially related to school or employment, asking for help (e.g., attending AA meetings), talking about challenges with other veterans, communicating needs to family members, having a place or support network to come home to, and making and maintaining connections. Specifically, our analysis revealed six personal experiential themes related to successful transition following deployment, including (1) Communication, (2) Having a shared experience with other vets, (3) Establishing a support network, (4) Creating stability and routine, (5) Asking for help, and (6) Productive coping.

#### 6.4.1 Communication

A common point of discussion was the importance of communication, particularly with family members. This finding helps to explain how participants made sense of their postdeployment experiences, particularly related to coping with relationship difficulty. Aaron shared, “But our biggest, the biggest help for transitioning is time and communication. Communicating with your spouse and understanding that this is the way things are. This is how we meld together. I think that helped in subsequent deployments for transitioning.” Earlier in his interview, Aaron mentioned participation in talk therapy, which may have helped with the development of individual communication skills and practices. However, this connection was not explicitly stated. Participants frequently highlighted the difficulty of finding their sense of belonging within their family structure after returning home from deployment, which emphasized the need for communication with family members. Jake's experience helps bring light to the role of communication in addressing this challenge, “You're trying to get back into your routine, to figure out your role as a man, as a father, as a husband, and they're trying to get into their routine as family, wife, husband, what have you, and [I] try to…identify any issues and problems firsthand to talk about them before they boil over to cause bigger problems.”

While communication with their family members was key to making a successful transition, participants also discussed the importance of sharing their experiences with others, particularly in contexts free of judgment (e.g., counseling). However, many participants disclosed their initial discomfort related to sharing these experiences, some noting that their participation in this research marked the first time they shared their stories with anyone. Daniel explained, “And then especially at first, because I wasn't talking to anyone about anything. And then I start talking to someone. And I was nervous because I felt like telling them would judge me for some of the things I was told…Now, that was my biggest thing about coming out, talking to people, was sharing my experience and my experiences and seeing that they judge me or not.” We learned that Daniel's experience was not uncommon as many of our participants expressed a sense of shame or fear related to their own deployment experiences. Yet talking about these experiences, in many ways, helped to diminish such destructive feelings. For instance, Aaron discussed how participating in this interview process was the first occasion he shared some of his experiences, “But, yeah, you are the first person I've told of big things. And [my wife] had told me…I need to put [my story] on paper, all the stuff I did…but I always start and I just never get back to it. So maybe this will give me my motivation now I'm talking to you about it.”

#### 6.4.2 Establishing a Support Network

In close parallel to sharing experiences with other vets, participants also highlighted the need to establish a support network even before coming home from deployment. Jessica explained, “…having a support network even during a deployment is important. Um. (She pauses) There is absolutely nothing you can do about most of the circumstances for the deployment, except for just do the job to the best of your ability and hope things work out for the best.” Members of a support network varied from one participant to the next, most included family members and other veterans, and some included the extended community. Megan shared how having a strong support network was already helping her with transitioning after deployment, “…The last two times when I transitioned, just because I already have friends in the area who have that support network built in…So I don't think it's going to be as [hard]. It's still going to be challenging, but I think it will be a little bit easier to at least try to settle on a civilian life for the next few years.”

In addition to having a sense of community support and understanding, many participants mentioned the role of their pets–especially dogs–in making sense of their reintegration experiences. Several participants even had dogs in the room while they completed their Zoom interviews. Thomas helped to explain how his dog assisted in a role that paralleled his support network, “And like even the dog would come up to me because he can tell I'm overwhelmed because I'm thinking about it. He's like, come on, pet me, like, you're fine.” The comforting and stabilizing effect of a support network cannot be over emphasized. It was frequently cited by participants as one of the single greatest contributors to their successful reintegration and was a large component in understanding how they make sense of these experiences. Overall, creating a support network and learning to rely on that network was a significant part of how participants made sense of their transition after deployment.

#### 6.4.3 Asking for Help

For nearly all of the participants, asking for help was paramount to their successful transition following deployment and was an important aspect in making sense of this process. Many participants expressed that coming home was the most difficult part of their entire deployment experience and highlighted how learning to ask for help is what allowed them to survive and move forward. Daniel shared, “Don't be afraid to ask for help. Take…anything that's offered for help. Take it into consideration. The worst thing that will happen is it won't work for you. But there's plenty of options. There's plenty of things to do. Talk to the older people who've been deployed. Talk to them…at least talk to someone. Because the hardest part is coming out [of deployment] …” Like other participants, Daniel openly discussed his participation in therapy, which likely influenced his own perspective about the importance of asking for help.

Several participants discussed how unhealthy coping strategies, such as alcohol consumption, created additional difficulties following deployment. Aaron, like others, disclosed the need to reach out for help through programs like Alcoholics Anonymous (AA), “And maybe it was one of the ways I was trying to cope with something. To be honest, I go to AA, and I'm trying to get myself better. You know, I've asked for help and sought help.” With many resources available to individuals returning from deployment today, one of the most important aspects that assisted participants in making sense of their reintegration experiences was learning to ask for help.

### 6.5 Growth Through Deployment Experiences

In addition to exploring the challenges and aspects that supported transition following deployment, the majority of participants shared accounts of personal growth that resulted, at least in part, from their deployments. While some individuals spoke of increased vigilance as a challenge, for instance, others discussed greater situational awareness as a means to being more perceptive and adaptable. Additionally, while many participants indicated that they would willingly deploy tomorrow if asked, a smaller number of participants were adamant that they would not participate in future deployments under any circumstances. Despite these differences, there were several similar points related to growth that the majority of participants shared as part of their sense‐making journey of reintegration. Megan's words help explain how this growth occurred, “As bad as this deployment was, I think it was very necessary and a good learning experience. I know I did grow a lot from it…” Megan was not alone in feeling this way, many participants expressed how they gradually developed valuable skills as a part of or following their deployment experiences. In all, we found six PETs of growth, including, (1) New perspective on and appreciation for life, (2) Empathy, (3) Appreciation and respect for cultural difference, (4) Adapting to cultural difference, (5) Resilience and adaptation, and (6) Leadership. Please note that while we elected not to discuss all six themes, the selected PETs are those that are most relevant to the present discussion regarding the sensemaking of MSMVs during reintegration.

#### 6.5.1 Empathy

A major point of discussion was participants' ability to gain new perspectives through their deployment experiences, which in turn, led to the development of greater empathy. Jessica explained, “Well, so in Iraq and Afghanistan, I saw the living conditions of the populations there, and I really did realize and come to understand how truly fortunate anyone who lives in the United States is.” Additionally, Joan, who worked as a nurse, shared how exposure to patients created greater understanding:

Well, a lot of that I have seen a lot of patients have to go through it, too. It made a bigger difference in understanding. I had sat with patients in the hospital before who were dying, but never in the aspect of like hospice working with them, helping them to address what they needed to address.

Both Jessica and Joan's accounts help demonstrate how they made sense of their experiences by choosing to view others with more compassion following their deployments. For many of our participants, making sense of their reintegration experiences focused on their own development of greater empathy for others.

#### 6.5.2 Appreciation and Respect for Cultural Difference

In making sense of their reintegration experiences, several participants talked about their view of cultural difference and how deployment helped them develop greater appreciation and respect for cultural practices divergent from their own. In fact, seven participants clearly articulated that their military and deployment experiences contributed to their own intercultural competence. It is worth noting that a smaller number of participants discussed how their deployments reinforced or resulted in feelings of dislike or even hatred for specific cultures different from their own. These contrasting experiences should be a point of investigation for future research.

As most participants agreed, Matthew shared how his appreciation for cultural difference increased through his experiences, “So I've come to appreciate…knowing that America is not the only way to do things, I guess. It's okay to do these other things that I've seen in other countries. So, I really respect a lot of things.” Aaron shared a similar account, explaining how his military service–particularly that which involved travel to other countries–helped him develop and grow:

I changed, you know…we talked about intercultural development…you know, I got thinking about it last night. My gosh, I had experienced all these different places, been to Morocco, to Spain, to Germany and so forth and so on, and experienced all these things, plus all this other stuff that I did. Yeah, it's changed who I was, because, of course, I was a young kid…and now it definitely impacted my life…I really want to couple this all together is through my military experience, I wouldn't be who I am right now.

Daniel expressed similar sentiments, sharing: “I kind of respect other cultures more and their practices, even though it's a lot different than ours. And we may not see it as correct in our eyes, but…they're doing what their culture usually does and…you see people from all over the world come together like it's just another day.” As these accounts help demonstrate, the majority of participants shared experiences of greater appreciation and respect for cultural difference as a part of their sense‐making process following deployment.

#### 6.5.3 Adapting to Cultural Difference

Yet another aspect of sensemaking involved the development of adaptability toward cultural difference. As with the previous theme, *appreciation and respect for cultural difference*, a majority of participants discussed this particular growth following deployment, though some did not. Megan described how, from her perspective, cultural adaptation was a form of respect: “It's probably had to do with my previous deployments…Just go somewhere and figure out how to be part of their culture, it's really not too hard…You adjust to that culture, at least somewhat…That's it for any culture, just be respectful.” In an adaptive example, Daniel described how, following deployment, he worked in a maximum‐security prison where he regularly interacted with Muslim prisoners and chose to handle religious items, such as prayer rugs or the Quran – the Islamic holy book – with great care and respect during security checks by wearing gloves to handle them or by having the prisoners move items themselves.

He shared, “I'd have them actually move [the Quran]…so I wouldn't touch it. I wouldn't touch their Quran, I'd have them open it, go through page by page, make sure nothing was in it. But I would…If I handled it, I'd have gloves on to respect their culture, their religion.” Daniel went on to explain the importance of this approach through his understanding of Islamic practices, “You don't want to, I guess taint it with anything…that may or may not be on your body. Like, say you were eating pork. You don't want to touch this stuff and have pork all of your hands because they don't eat pork.” These excerpts help demonstrate how participants made sense of their experiences following deployment by adapting to cultural difference, a valuable skillset, which is further investigated in the discussion portion of this paper.

#### 6.5.4 Resilience and Adaptation

Another way that participants made sense of their reintegration experiences following deployment was through their ability to develop resiliency and to adapt to new circumstances and situations. Megan shared, “I think I became a lot more resilient out there, a lot tougher. I don't let stuff bother me near as much as I used to…just a lot more accountable for my actions and what I do.” Others noted an increased sense of personal accountability and responsibility in making sense of their postdeployment experiences. Participants also discussed how they developed skills for making critical decisions in stressful situations and often described themselves as being more action‐oriented (e.g., responsive in an emergency situation) following their deployment experiences. In making sense of their reintegration experiences, participants often discussed how they developed more resiliency and adaptability following deployment. In some cases, their actions led to life‐saving interventions, which helps underscore the value of this investigation and a potential area of focus for future research.

## 7 Discussion

The purpose of the present IPA study was to investigate how MSMVs make sense of the transition home after deployment. Notably, the findings of this IPA study revealed three categories of PETs, including transitional challenges, transitional support aspects, and growth through deployment experiences. Findings suggest that MSMVs face myriad transitional challenges (e.g., relational turbulence) when seeking to reintegrate into civilian life following deployment. Their challenges appear to be exacerbated by complex trauma, grief, and loss. These findings serve to supplement existing literature related to challenges of MSMVs as they seek to reintegrate into society (Gettings et al. 2019; Orazem et al. 2017; Rossetto 2015; Sayer et al. 2011b).

A unique contribution of this study are the findings related to intercultural competency, specifically, *Appreciation and Respect for Cultural Difference* and *Adapting to Cultural Difference*. While experiences of PTG among MSMVs are well‐documented (e.g., Mark et al. 2018; Parrish‐Sprowl et al. 2014; Tsai and Pietrzak 2017; Tsai et al. 2015), specific growth related to intercultural competency has not been clearly established in past research. Work by Buechner (2020) and Oliva (2022) proposed that intercultural competency or a global mindset may provide meaningful skills for successful transition to civilian life following service. As we discussed in the findings, most but not all participants described growth related to intercultural competency. We recommend that future researchers further investigate the development of intercultural competency among MSMVs, particularly using quantitative measures which could help explain factors that contribute to the development of intercultural competency.

When considering the prevalence of relational uncertainty in the sensemaking of reintegration for MSMVs, there is merit in further connections being made to the relational turbulence model (RTM). Introduced by Solomon and Knobloch (2004), this model contributes to our understanding of the ways in which romantic partners navigate relational uncertainty. This model illustrates the ways in which relationships have been found to be affected during complicated processes like reintegration (Theiss & Knobloch 2013). Based on the results of this IPA study, it becomes clear that RTM may be combined with future research aimed at assisting reintegration sensemaking for service members.

As a recommendation to those institutions interested or invested in the successful reintegration of military members, the emphasis of communication pattern development should not be overlooked. The findings of the present study indicate that the sensemaking of these participants related most regularly to their communication whether intrapersonal or interpersonal in nature. Previous research has been helpful in identifying relevant communication patterns that may assist in the support of military personnel following deployment. Gettings et al. (2019) explored which messages contribute to or frustrate support‐seeking for veterans asserting the priority of, “communicating acceptance and autonomy support while encouraging behavioral health care help‐seeking” (Gettings et al. 2019, p. 221). Knowledge of this model and the findings of this present study may benefit reintegration‐supportive organizations with greater priority on communication development and supportive messaging. Institutions may also find it beneficial to explore those resources which might be provided in support of MSMV's before and during active‐duty deployment situations. Such resources may provide additional support to these individuals as they seek to make sense of these uniquely demanding experiences.

This study introduces an important conversation about the possible practical implications through better understanding of the sensemaking among MSMVs as they reintegrate into a domestic/civilian context. The idea of “coming home” stands out prominently in the voice of the participants and made clear the sharp contrast in life experience for participants from their deployment time to their transition “home.” The contrast of these experiences were sharp in the voice of the participants as it was in the sensemaking they employed to account for this life altering process. These findings might alert MSMVs, and their respective support system, to the need for shared communication patterns directed to dialog about this process of coming home (Gettings et al. 2019). The recurrent themes of this study highlight the relational turbulence common to this population and may assist policy decisions and legislations aimed at supporting our U.S.13, no. veterans (Solomon & Knobloch 2004; Theiss & Knobloch 2013). Supportive communication for MSMV's has been confirmed as a merited area of discussion in practical application, institutional policies, and theoretical exploration.

## 8 Limitations and Directions for Future Research

The purpose of qualitative research is not to be widely generalizable (Korstjens and Moser 2017). However, we introduced multiple validation strategies that are intended to help readers draw their own conclusions about the transferability of these findings. Additionally, we suggest that the emergence of themes in the present study suggests some commonality of shared experiences related to participant challenges, support aspects, and growth following deployment despite the diversity of participants' specific deployment experiences. A noted limitation of this study is the recruitment of participants from diverse political, cultural, and social contexts related to the war, potentially influencing the reintegration experiences based on varying socioeconomic backgrounds. Moreover, this recruitment approach may have overlooked the impact of the macro environment on the quality of micro‐level experiences for respondents, their families, and other key social networks during reintegration. Future research should include more limiting recruitment criteria that would help account for such nuances.

Another limitation is the wide spanning age difference across participants since individuals may have different experiences depending on the time frame in which they were deployed and the reintegration resources available at the time. While the recency of deployment varied for the participants, there is evidence to suggest the merit of utilizing IPA to explore reintegration experiences even if significant time has passed between the deployment and the interview or data collection (McCormack & McKellar 2015). Farr and Nizza reinforce this stating, “when a critical event is very traumatic, investigating it longitudinally even after some time has passed can be meaningful” (Farr and Nizza 2019, p. 206). Even so, the recency of their experiences may influence the participants' memory recall. Thus, future studies may focus on participants who have served a recent term of deployment.

Moreover, we did not ask participants specifically about combat experiences for two reasons: (1) to help minimize re‐traumatization of our participants and (2) because the focus of this research was on how participants make sense of coming home following deployment (not the deployment itself). We acknowledge that participants likely had very different experiences depending on their environment, whether through battle‐involving deployment or peacetime deployment, and that these experiences may have impacted the success of their reintegration following deployment. We also acknowledge that the wide range of deployment experiences (e.g., combat vs. noncombat zones, length of time, location of deployment) is a limitation of this study. More specifically, we expect that the findings of this study would likely highlight aspects related to stress and trauma (among other psychological factors) if all participants had been deployed in active combat zones, and that their transition would have included additional challenges (e.g., coping with physical injury, death of a comrade(s), survivor's guilt, etc.) beyond those which emerged in the present study. Therefore, future research should focus on populations with similar deployment factors, such as length of time deployed and whether the deployment occurred in a combat zone.

Another limitation of this study is the homogeneity of participants in terms of race and ethnicity, given that 90% of our participants self‐identified as White. Future studies should work to investigate the experiences of individuals, particularly among more marginalized populations, since their experiences are likely to be divergent, in some ways, from those discussed here. For instance, we anticipate that greater challenges may be faced in trying to access resources following deployment among marginalized populations, in particular members of underrepresented racial identities and the LGBTQ community. We also acknowledge that while we interviewed six Active‐Duty members and four Guard/Reserve members for this study, we did not specifically ask about the differences between their and their family members' access to resources before, during, and after deployment. Nor did we specifically ask about access to technology and in‐person visits during these deployments. Since access to such resources and opportunities may have impacted the outcome of individual deployment experiences, we suggest that this is another area of focus for future research.

Moreover, while we learned about the importance of communication and asking for help as strategies that aided the resettlement process following deployment, we did not explicitly ask how our participants developed these skills or from where this knowledge stemmed (e.g., talk therapy, existing military resources and programs). Investigating the source of such knowledge and skills in future studies could be particularly helpful for better understanding gaps in existing resources and programs (i.e., what is working well already and what is not). Finally, we suggest that future quantitative research investigate the impact of potentially related factors on reintegration experiences, including (1) service members by military component (Active Duty, Reserve or National Guard), (2) their personal living situation (e.g., returning to a significant other, returning to a family routine, etc.), and (3) their employment status (e.g., returning with a unit and to military status, returning to a civilian employer, returning to no employment) since these variables are likely to have a measurable impact on one's reintegration experiences.

While we acknowledge that there are many challenges regarding the diversity of participants' individual demographics and deployment experiences, we note that the focus of the present study was on reintegration experiences *following* deployment, not the deployment itself. Moreover, nine out of 10 participants completed at least one international deployment, and eight out of 10 participants completed at least one deployment period lasting 6 months or longer, which supports some deployment commonalties. Finally, participants voiced common experiences during reintegration including challenges, support aspects, and growth which was evidenced by emergent themes that represented the majority of participant accounts.

## 9 Concluding Thoughts

In summary, the current study provides important insights into how MSMVs make sense of their reintegration experiences following deployment through exploration of transitional challenges, transitional support aspects, and growth through deployment experiences, as well as the urgent need for comprehensive community‐based, growth‐focused initiatives to support their reintegration into civilian communities. Such initiatives may provide important implications for developing suicide awareness and prevention campaigns for veteran populations. Despite many challenges, the veterans in this study possessed valuable skills and insights, enhanced by their connection to others. We hope that this paper generates future research and the development of new programs, which recognize that “coming home is the hardest part” and focus on aspects that can help assist with transition and the potential for growth through these experiences.

## Conflicts of Interest

The authors declare no conflicts of interest.

### Peer Review

The peer review history for this article is available at https://www.webofscience.com/api/gateway/wos/peer-review/10.1002/jcop.23178.

## Data Availability

The participants of this study did not give written consent for their data to be shared publicly, so due to the sensitive nature of the research supporting data is not available.
